# A New Calibration Circuit Design to Reduce Drift Effect of RuO_2_ Urea Biosensors

**DOI:** 10.3390/s19204558

**Published:** 2019-10-20

**Authors:** Po-Yu Kuo, Zhe-Xin Dong

**Affiliations:** Graduate School of Electronic Engineering, National Yunlin University of Science and Technology, Douliu 64002, Taiwan; M10713240@yuntech.edu.tw

**Keywords:** urea biosensor, ruthenium oxide (RuO_2_), drift effect, calibration circuit

## Abstract

The goal of this study was to reduce the drift effect of RuO_2_ urea biosensors. A new calibration circuit (NCC) based on the voltage regulation technique with the advantage of having a simple structure was presented. To keep its simplicity, the proposed NCC was composed of a non-inverting amplifier and a voltage calibrating circuit. A ruthenium oxide (RuO_2_) urea biosensor was fabricated to test the calibrating characteristics of the drift rate of the proposed NCC. The experiment performed in this study was divided into two main stages. For the first stage, a sound RuO_2_ urea biosensor testing environment was set-up. The RuO_2_ urea sensing film was immersed in the urea solution for 12 h and the response voltage was measured using the voltage-time (V–T) measurement system and the proposed NCC. The results of the first stage showed that the RuO_2_ urea biosensor has an average sensitivity of 1.860 mV/(mg/dL) and has a linearity of 0.999 which means that the RuO_2_ urea biosensor had been well fabricated. The second stage of the experiment verified the proposed NCC’s functions, and the results indicated that the proposed NCC reduced the drift rate of RuO_2_ urea biosensor to 0.02 mV/hr (98.77% reduction).

## 1. Introduction

Recently, cases of kidney diseases have been increasing due to changes in diet and eating patterns. Urea plays an important role in clinical processes for detecting kidney functions [1]. For the past decades, many urea biosensors have been widely studied to achieve high sensitivity, linearity, and some sensing characteristics [1,2,3,4,5,6,7,8,9,10,11,12,13,14,15,16,17,18,19,20,21,22,23,24,25]. However, biomedical workers still find the current biosensors that measure drift rates unacceptable and unsatisfactorily. Scientists suspected that the unstable readout of urea biosensor for long-term measurement may be due to the drift effect. During long-term measurement, the response voltage of the sensor is changing with time, which is called the drift phenomenon. The reason that response voltage change is that hydration layer formed on the surface of sensing film [26,27]. The hydroxyl groups were formed on the surface of sensing film in solution, and the hydrated ions, formed by coulombic attraction between water molecules and ions, diffused to the sensing film will result in the formation of hydration layer. The surface potential of the film was attributed to electrical double layer capacitance which was formed by the hydration layer. Hence, some researchers devoted themselves to finding new biosensor materials. At present, the most widely used sensing film in fabricating urea biosensors is composed of nickel oxide (NiO) and titanium oxide (TiO_2_). The NiO has strong chemical stability and fast electron transfer capability; therefore, it is also applied as a sensing material to develop uric and glucose sensors [1,5,6,7]. TiO_2_ is a non-toxic, non-corrosive, and reusable material with better electron transition, making it a good sensing film material for biosensors [8,9,10,11,12]. Ruthenium oxide (RuO_2_) is a transition metal oxide with rutile-type structure and high metallic conductivity; RuO_2_ is a suitable material for working electrodes due to its low resistivity, high thermal stability, and good diffusion barrier properties [28,29,30,31]. In previous studies, RuO_2_ has been applied as a sensing material to fabricate pH [31] and chloride sensors [32,33]. The above researchers also demonstrated that the proposed sensors achieved better sensing properties such as average sensitivity and linearity. Therefore, RuO_2_ is a good material for fabricating biosensors. According to the above discussions, many researchers apply NiO and TiO_2_ as materials to improve the sensing characteristics of urea biosensors. However, the drift effect is rarely discussed in these articles. Recently, Chou et al. [27] presented urea biosensors based on graphene oxide/titanium dioxide films modified by urease-magnetic beads to achieve better sensing characteristics. The drift problem is still not solved in this reported work. 

Although different urea biosensors had been widely studied to achieve better sensing characteristics [1,2,3,4,5,6,7,8,9,10,11,12,13,14,15,16,17,18,19,20,21,22,23,24,25], the non-ideal effects and drift rates had been barely discussed in previous works. Chou et al. [24] presented a flexible arrayed urea sensor based on urease-magnetic beads (MBs) and graphene oxide (GO), but it was only able to measure the drift rate and the drift effect calibration method was not discussed. This non-ideal effect is critical for biosensors and must be resolved [32,33].

In this study, RuO_2_ was used as a sensing film to fabricate a urea biosensor, and a new calibration circuit (NCC) was proposed to reduce the drift effect of an RuO_2_ urea biosensor. To verify the functions of the RuO_2_ urea biosensor, several sensing characteristics were measured within the normal urea concentration range of the human body (2.5–7.5 mM) [25], using the conventional voltage–time (V–T) measurement system. Based on the results, with the application of the voltage regulation technique, the proposed NCC obtained a significantly lowered drift rate compared to that of the conventional V–T measurement system. Moreover, the sensing characteristics of the RuO_2_ urea biosensor were also compared to those measured by the noise-canceling readout circuit proposed in Kuo’s work [34]. In this reported work, a new readout circuit was presented to reduce the power line noise during sensing measurements of urea biosensors. In Kuo’s work, the proposed circuit, a Twin-T notch filter was used to cancel the power line noise, and a Sallen–Key low-pass filter was used to suppress the high-frequency noise. However, this readout circuit only improved the two sensing characteristics, average sensitivity and linearity. The sensing characteristics in [34] were also compared with those of the proposed urea biosensor in this work, and our RuO_2_ urea biosensor achieved better average sensitivity and linearity. 

## 2. Experiment

To realize the study goals, the experiment was divided into two stages: the first stage of the experiment entailed setting-up of a sound RuO_2_ urea biosensor testing environment, and the second stage involved verification of the proposed NCC’s functions. For the convenience of the readers, this section was presented in detail in terms of materials, manufacturing of the flexible arrayed RuO_2_ urea biosensor, the V–T measurement system, and the proposed NCC for drift rate.

### 2.1. Materials

The polyethylene terephthalate (PET) substrate used in this study was purchased from Zencatec Corporation (Tao-Yuan City, Taiwan). The required ruthenium (Ru) purity was targeted at 99.95% and it was sourced from Ultimate Materials Technology Co., Ltd (Hsinchu County, Taiwan), which deposits ruthenium dioxide (RuO_2_) film onto PET substrate by the sputtering system. Besides, silver was used in this study as electrodes made from arrayed wires, which was purchased as silver paste from Advanced Electronic Material Inc. (Tainan City, Taiwan), and was made into silver wires using a screen-printing system. This study also employed an epoxy thermosetting polymer (product no. JA643) from Sil-More Industrial, Ltd. (New Taipei City, Taiwan) cured using a screen-printing technology to make an insulation layer. Urease and urea were purchased from Sigma-Aldrich Corp. (St. Louis, MO, USA) and J. T. Baker Corp. (St. Louis, MO, USA), respectively. Phosphate monobasic (KH_2_PO_4_) powders and potassium phosphate dibasic (K_2_HPO_4_) powders were purchased from Katayama Chemical Industries Co., Ltd. (Yasuo Machi, Japan), and were used to make 30 mM phosphate buffer saline solutions (PBS) with a pH level of 7 (considered neutral in the human body). The deionized (D.I) water, which was approximately equal to 18.4 MΩ cm^−1^, was used to prepare the aqueous solutions.

### 2.2. Manufacturing of the Flexible Arrayed RuO_2_ Urea Biosensor 

**Figure 1:**
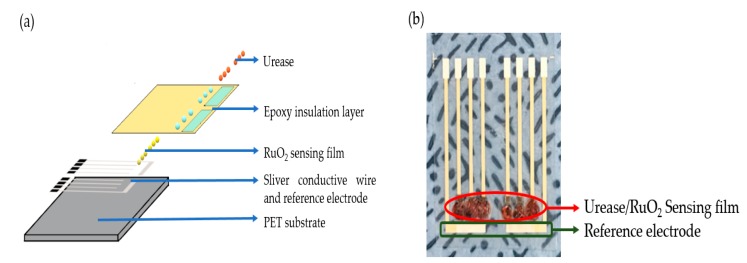
(**a**) The structure diagram of the flexible arrayed RuO_2_ urea biosensor, and (**b**) top view of the completed flexible arrayed RuO_2_ urea biosensor.

RuO_2_, a transition metal oxide with high-temperature stability, low resistivity, and good diffusion barrier properties was used in this study to fabricate the flexible arrayed urea biosensor. The production process of flexible arrayed RuO_2_ urea biosensor was similar to Chou’s previous work [24]. The structure diagram of the flexible arrayed RuO_2_ urea biosensor is shown in Figure 1. 

The manufacturing process of the flexible arrayed RuO_2_ urea biosensor started with the silver paste being printed on flexible arrayed PET substrates using screen printing techniques to form the arrayed silver wires producing the working electrode and the reference electrode. Next, the RuO_2_ film was deposited on the flexible arrayed PET substrate through a sputtering system to form the RuO_2_ film window; then, it was encapsulated with an epoxy thermosetting polymer. The urease was immobilized because of the covalent bond, its low diffusivity, and strong binding, which reduced the loss and stability of the urease [25]. Afterward, the aminopropyltriethoxysilane (APTS) solution was dropped on the RuO_2_ sensing film at room temperature. The ability of urease to be adsorbed on the surface was enhanced by dropping 1% glutaraldehyde solution onto the RuO_2_ sensor which was kept still for 24 h. Finally, the urease was dropped onto the RuO_2_ sensing film to form a flexible arrayed RuO_2_ urea biosensor.

### 2.3. V–T Measurement System

**Figure 2:**
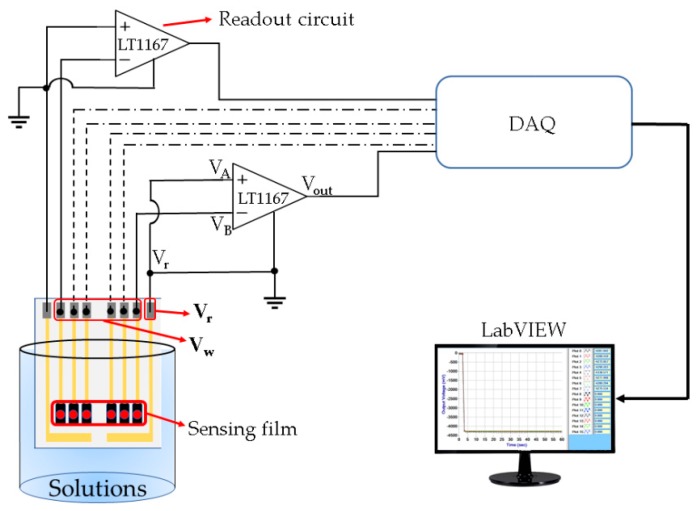
The schematic diagram of the traditional V–T measurement system [32].

**Figure 3:**
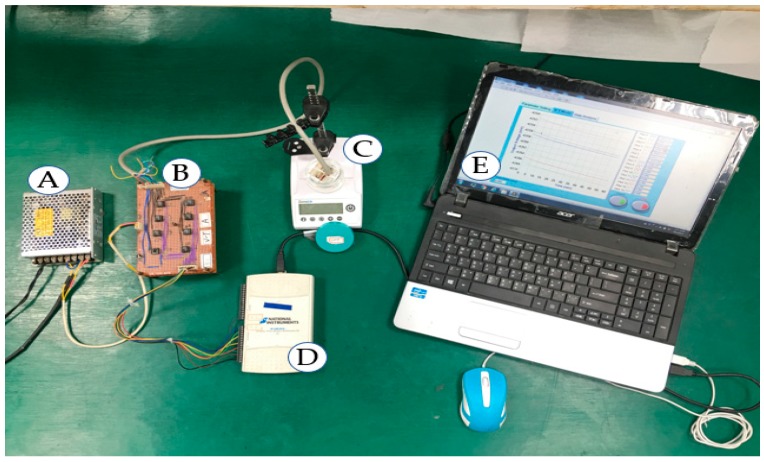
The actual V–T measurement system. (**a**) DC power supply. (**b**) Readout circuit. (**c**) Urea solution and urea biosensor. (**d**) Data acquisition (DAQ) device. (**e**) Computer.

The sensing characteristics of the flexible arrayed RuO_2_ urea biosensor was measured using the V–T measurement system. The V–T measurement system had been reported in previous studies [32,33]. The measurement system consisted of an LT1167 instrumentation amplifier (Type: LT1167CN8#PBF, Linear Technology/Analog Devices Crop., Norwood, MA, USA), a data acquisition (DAQ) device (Type: USB-6210, National Instruments Crop., Austin, TX, USA), and a program system software (Type: LabVIEW, National Instruments Crop., Austin, TX, USA). The schematic diagram and the actual V–T measurement system used are shown in Figure 2 and Figure 3, respectively. In Figure 3, the V–T measurement system was composed of a power supply, a readout circuit (LT1167), a data acquisition card (DAQ device, Model: NI USB-6201, National Instrument Corp., Austin, TX, USA), and a computer with LabVIEW program [32,33]. The response potential was sensed by the readout circuit. To obtain the digital signals, the output voltage will be transmitted to the DAQ device. Finally, the digital data were transmitted into the computer and analyzed by the LabVIEW program. The LT1167 instrumentation amplifier is a precision electronic component with a high input impedance of about 200 GΩ, which means that the input signal will not be attenuated; the disadvantage of this is that the input noise may be amplified resulting in unstable measurement results. The LT1167 instrumentation amplifier has the advantage of a higher common-mode rejection ratio (CMRR) (90 dB, G = 1) and power supply rejection ratio (PSRR) (105 dB, G = 1), which means that the amplifier is effective in resisting noise and power noise. The LT1167 instrumentation amplifier’s gain was set between 1 (0 dB) to 1,000 (60 dB), which was controlled by an external resistor. The LT1167 instrumentation amplifier was used to read the response voltage of the biosensor. The output voltage and gain were computed as:

(1) $$\mathrm{Gain}\;\left(G\right)=1+\left(\frac{49.4k}{R_{G}}\right)$$

(2) $$V_{out}=\left(V_{A}-V_{B}\right)\times \left(1+\frac{49.4k}{R_{G}}\right)=\;V_{r}-V_{W}=\;-V_{w}\left(R_{G}\approx \infty \right)$$

where:

*V_out_* is the response voltage of the LT1167 readout sensor,
*V_A_* is the positive input of the amplifier,
*V_B_* is the negative input of the amplifier,
*R_G_* is the external resistor,
*V_r_* is the voltage of the reference electrode,
*V_w_* is the voltage of the working electrode.

In this V–T measurement system, the internal resistance was set at 49.4KΩ, the external resistor *R_G_* controlled the magnification of the LT1167 instrumentation amplifier, and the biosensor was immersed in the urea solution. Based on Equation (2), the gain of the LT1167 was set to 0 dB, so *R_G_* does not need a separate external resistor to amplify the voltage gain; R_G_ was regarded as an open circuit (*R_G_* is infinite). Furthermore, *V_out_* represents the potential difference between the reference electrode and the working electrode. Since the reference electrode was connected to the ground (*V_r_* = 0 V), the response voltage of the RuO_2_ urea biosensor was −*V_w_*. The response voltage was then captured by the DAQ device, and the analog signal was converted into a digital signal, which was transmitted to the LabVIEW software program to display the sensing voltage of the biosensor on the computer.

### 2.4. The Proposed New Calibration Circuit for Drift Effect

**Figure 4:**
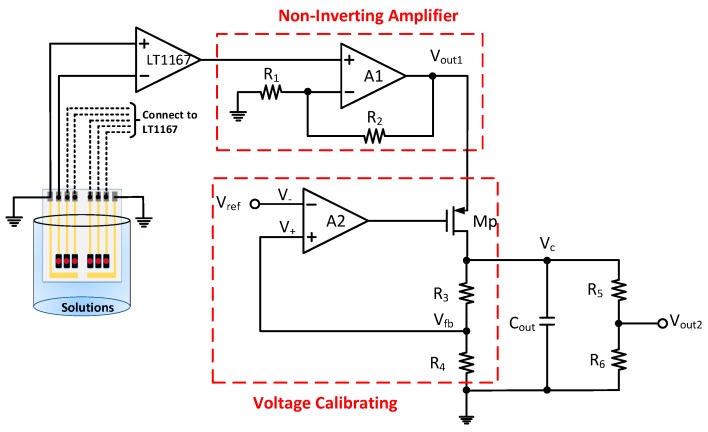
The structure diagram of the proposed new calibration circuit (NCC).

**Figure 5:**
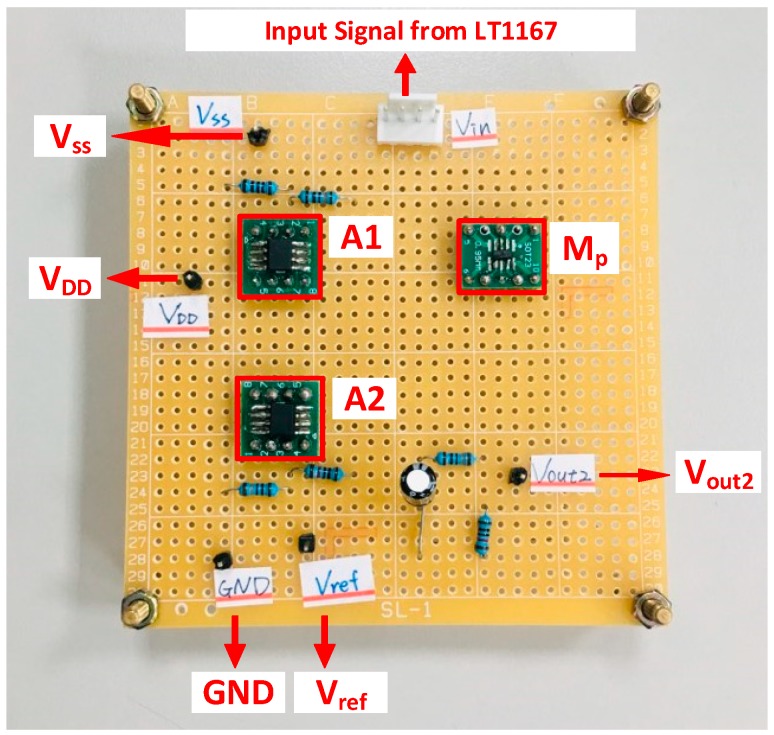
The implementation of the proposed calibration circuit.

A new calibration circuit (NCC) was proposed by this study to reduce the drift effect of the flexible arrayed RuO_2_ urea biosensor as shown in Figure 4. This NCC consisted of a non-inverting amplifier (A_1_, R_1,_ and R_2_) and an error amplifier A_2_, a P-MOSFET transmission transistor M_p,_ a negative feedback network (R_3_ and R_4_), an output voltage capacitor C_out_, and a resistor divider (R_5_ and R_6_). The implementation of the proposed NCC is shown in Figure 5. The operational amplifier OPA130 was used as the error amplifier A_1_ and A_2_. A P-Chanel MOSFEF FDN340P-ND was utilized as the drive transmission transistor M_p_. The proposed circuit was attached to a real circuit board with simple discrete devices such as resistors, capacitors, field-effect transistors, and amplifiers for its simplicity. The operational amplifier OPA130 is a low-power precision FET-input operational amplifier, which has a higher CMRR (90dB min) and has good noise resistance. It also has a wide voltage supply range (V_+_ & V_-_ = ±2.25 V to ±18 V), which means that the non-inverting and the inverting inputs of the OPA130 have a wider range. The amplifier magnification factor was improved by controlling its bias in the non-saturation mode. The low response voltage of the biosensor was amplified by the non-inverting amplifier, enabling transmission of the transistor M_p_ in the linear region. M_p_ provided output current to charge the output load capacitor (C_out_) maintaining *V_c_* at a high level. The mechanism of the voltage regulation is depicted as follows: it is implemented by the negative feedback resistors (R_3_ and R_4_), and the feedback voltage network is expressed as the formula in Equation (3).

(3) $$V_{fb}=V_{c}\times \frac{R_{4}}{R_{3}+R_{4}}$$

Based on Equation (3), when the *V_c_* output voltage is decreased, the *V_fb_* will also decrease compared to the reference voltage V_ref_. In this period, the voltage difference between the V_+_ terminal and the V_–_ terminal of the error amplifier will be reduced, and the gate voltage of M_p_ will also be reduced. Therefore, the source-gate voltage (V_gs_) of M_p_ will increase and will generate more current to charge the output load capacitor. The output voltage V_c_ will then increase and remain at a stable level. A high *V_c_* voltage will result in an increased output voltage of the error amplifier and decreased the source-gate voltage (V_gs_) of M_p_. Under this condition, M_p_ will reduce the current to charge the output load capacitor. In this way, the output voltage will be controlled at a stable level as well. By changing the voltage or current, the output voltage can be maintained at a fairly stable level in a very short period.

The response voltage of RuO_2_ urea biosensor was read by LT1167, and the non-inverting amplifier (A_1_, R_1_ and R_2_) was used to amplify the sensing voltage which was input into the voltage calibration circuit. To obtain the real response voltage, the original magnification factor was reduced through the voltage divider (*R*_5_ and *R*_6_). The output voltage *V_out2_* was calculated using Equation (4).

(4) $$V_{out2}=V_{c}\times \frac{R6}{R_{5}+R_{6}}$$

The drift rate is a non-ideal effect in a biosensor during the measurement process. During long-term measurement, the response voltage of the sensor will gradually reach a stable level; this is called the drift rate [32,33,35]. In practice, the response voltage slowly changes proportionally with time during long-term measurement. Most researchers believe that this as a tedious and an unsolved issue. Based on previous studies [32,33,35], the response voltage of sensors stabilizes after 5 h. The drift rate can be calculated using Equation (5).

(5) $$\mathrm{Drift}\;\mathrm{Rate}=\frac{\mathrm{Drift}\;\mathrm{Voltage}}{\mathrm{Time}}=\frac{V_{12th}-V_{5th}}{7\;\left(\mathrm{hours}\right)}$$

The drift rate in this study was obtained within different time frames which were between the response potential of output node at 5th and 12th hour. The values obtained were then divided by the measured time intervals (7 h). To maintain the response voltage of urea biosensor at a stable level during long-term measurement, the proposed NCC was applied to measure the response voltage. The response voltage was controlled at a stable level by the proposed NCC, which then significantly reduced the drift rate. 

## 3. Results and Discussion

### 3.1. Sensing Properties of the Flexible Arrayed RuO_2_ Urea Biosensor

In this study, urease was used as the enzyme electrode. Urea was catalyzed and hydrolyzed by the urease and the reaction can be expressed as follows:

(6) $$NH_{2}CONH_{2}+3H_{2}O\overset{Urease}{\to }2NH_{4}^{+}+HCO_{3}^{-}+OH^{-}$$

**Figure 6:**
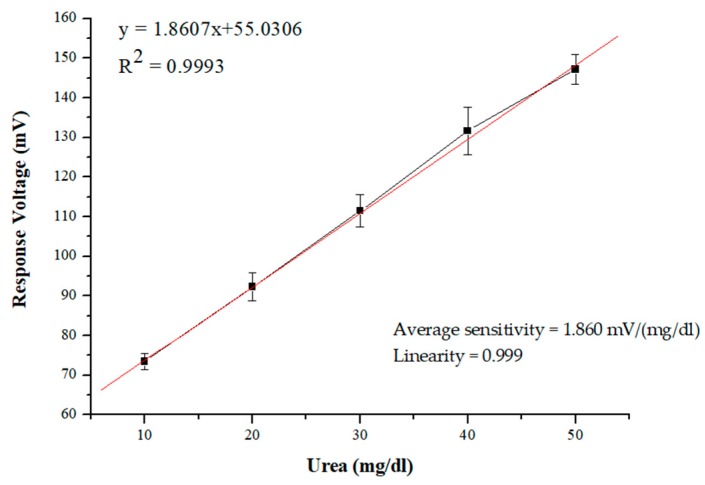
The results of the sensing measurement of the flexible arrayed RuO_2_ urea biosensor.

In this chemical equation, urea reacts with urease and is converted to ammonium (NH4^+^), bicarbonate (HCO_3_^−^), and hydroxide ions (OH^−^) [25]. The sensing properties of the flexible arrayed RuO_2_ urea biosensor were measured by the V–T measurement system. The flexible arrayed RuO_2_ urea biosensors were immersed in 10, 20, 30, 40, and 50 mg/dL urea concentration solutions. The output voltages were measured by the V–T measurement system five times. The results of the sensing measurement are shown in Figure 6. The average sensitivity and linearity recorded were 1.860 mV/(mg/dL) and 0.999, respectively. Moreover, the limit of detection (LOD) was estimated in this study. Limit of detection (LOD) is defined as the lowest concentration of analyte that can be detected in the analyte, but not quantitated [36]. In this experiment, a RuO_2_ urea biosensor was immersed in a buffer solution, and the LOD was measured by a V–T measurement system. To obtain the analyte concentration of the LOD urea biosensor, we followed the three sigma rule [37] and the equations are shown as follows:

(7) $$\sigma =\;\sqrt{\frac{\sum^{\text{​}}{\left|x-\bar{x}\right|}^{2}}{n}}$$

(8) $$\mathrm{LOD}=\frac{3\sigma }{S_{0}}$$

where σ is the standard deviation of measuring the response voltage of the PBS solution, x is value in the data, $\bar{x}$ is the mean of the data, *S*_0_ is the sensitivity. The experimental results showed that the response voltage of the PBS solution was −7.348 mV, the standard deviation of σ is 0.807 and *S_0_* is 1.860 mV/(mg/dL). Hence, the detection limit was calculated as 1.345 mg/dL. The sensing characteristics of the flexible arrayed RuO_2_ urea biosensors are shown in Table 1. Table 2 shows the comparisons of urea biosensors with various sensing films. 

### 3.2. Analysis of the Response Time of the Flexible Arrayed RuO_2_ Urea Biosensor

**Figure 7:**
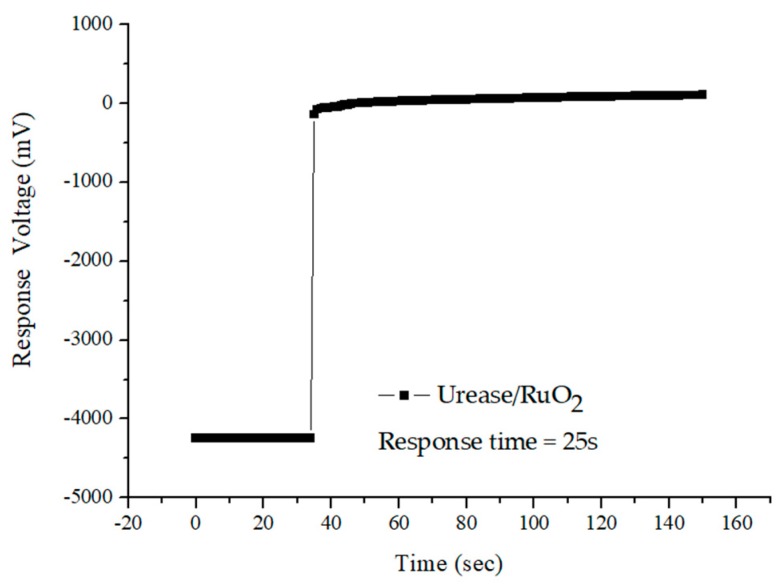
The response time of the flexible arrayed RuO_2_ urea biosensor.

To measure the response time of the urea biosensor, the RuO_2_ urea sensor was immersed in 30 mg/dL urea solutions. The response voltage of the sensor did not change immediately when the input potential signal change occurred. It required some time to change from origin state to a stable state. The response time is defined as the time required for the sensor output to change from its original state to 95% of its equilibrium value [24]. In this study, the response time of the flexible arrayed RuO_2_ urea biosensor was measured by the V–T measurement system. The result of the experiment’s response time is shown in Figure 7. The RuO_2_ urea biosensor exhibited a fast response time of 25 s.

### 3.3. Analysis of Interference Effect of the Flexible Arrayed RuO_2_ Urea Biosensor

**Figure 8:**
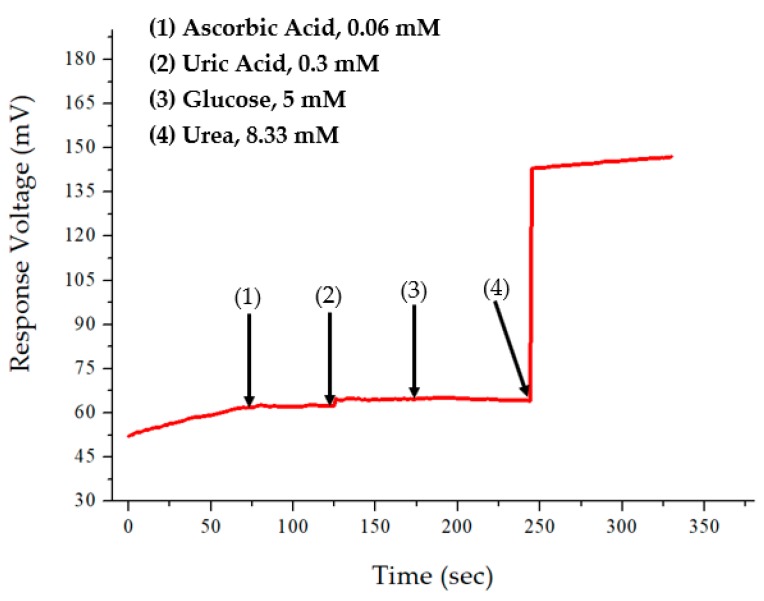
The result of the interference effect for flexible arrayed RuO_2_ urea biosensor.

The interference effects could determine the type of urea biosensor to be used. The selectivity of the urea biosensor is related to the analyte measurement, and the potential interference for the human body could be uric acid, ascorbic acid, and glucose [27]. For the experiment, interferences were added in the measurement to determine the performance of the urea biosensor and to test the selectivity of the RuO_2_ urea biosensor. During practical application, the interference solutions were selected based on the normal range of the solutions obtained on the sample human blood. The urea sensor was first immersed into the test urea solution 1.66 mM (10 mg/dL); then, the ascorbic acid solution (0.06 mM), uric acid solution (0.3 mM), and glucose solution (5 mM) were added into the test solution, sequentially. Finally, the 8.33 mM (50 mg/dL) urea solution was added to the test solution. The results of the interference effect are shown in Figure 8. It was observed that the flexible arrayed RuO_2_ urea biosensor was not easily interfered with by other materials. 

### 3.4. Analysis of the Drift Effect of the of the Flexible Arrayed RuO_2_ Urea Biosensor

**Figure 9:**
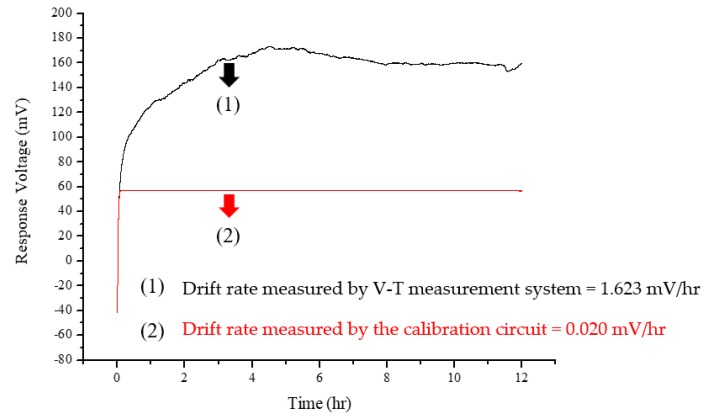
The drift effect experiment results in the flexible arrayed RuO_2_ urea biosensor.

As mentioned, the drift rates in this study were obtained within different time frames which were between the response potential of output node at 5th and 12th h. The result was then divided by 7 h which was the measured time interval. The drift rate, as previously discussed, was calculated using Equation (5). In the drift effect experiment, the RuO_2_ urea biosensor was immersed into the 40 mg/dL urea test solution for 12 h. The drift effect of the flexible arrayed RuO_2_ urea biosensor was tested by first, measuring the drift voltage through the conventional V–T measurement system to observe the response-voltage characteristics for 12 h; and by measuring the same RuO_2_ urea biosensor using the proposed NCC. From the experimental results shown in Figure 9, the drift rate measured by the V–T measurement system was 1.623 mV/hr. By applying the NCC, the drift rate was reduced to 0.02 mV/hr. These experimental results indicate that the proposed NCC reduced the drift rate obtained by the V–T measurement system by 98.77%. This study also compared the drift rate obtained by NCC with that of previous studies and is shown in Table 3.

### 3.5. Analysis of the Hysteresis Effect of the of the Flexible Arrayed RuO_2_ Urea Biosensor

**Figure 10:**
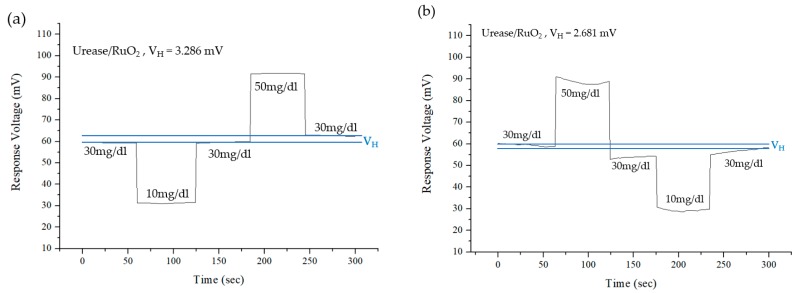
Hysteresis effect of the flexible arrayed RuO_2_ urea biosensor. (**a**) 30 mg/dL→10 mg/dL→.30 mg/dL→50 mg/dL→30 mg/dL and (**b**) 30 mg/dL→50 mg/dL→30 mg/dL→10 mg/dL→30 mg/dL.

Traditionally, biosensor devices’ readout results are usually recorded more than twice, as with the RuO_2_ urea biosensor. The concentration of the test solution could be changed after resumption originally buffers solution. There might be a voltage difference during the second time measurement process. This is called the hysteresis voltage [26]. Typically, the hysteresis voltage is due to the change of response potential. The hysteresis effect can be measured by immersing RuO_2_ sensing films in the loop of urea solutions for 60 s in different urea solutions. Then, the difference of initial and last response voltages of the same urea solution is called hysteresis voltage [26]. The hysteresis effect was measured by immersing the flexible arrayed RuO_2_ biosensor in a urea solution in the loops of 30 mg/dL → 10 mg/dL → 30 mg/dL → 50 mg/dL→ 30 mg/dL and 30 mg/dL → 50 mg/dL → 30 mg/dL → 10 mg/dL → 30 mg/dL, respectively. Figure 10 shows the hysteresis effects of flexible arrayed RuO_2_ urea biosensor. From the results, the hysteresis voltages of the RuO_2_ urea biosensor in the loop 30 mg/dL → 10 mg/dL → 30 mg/dL → 50 mg/dL→ 30 mg/dL was 3.286mV. The hysteresis voltages of the RuO_2_ urea biosensor in the loop 30 mg/dL → 50 mg/dL → 30 mg/dL → 10 mg/dL → 30 mg/dL was 2.681 mV.

## 4. Conclusions

This paper proposed a new calibration circuit (NCC) to reduce the drift rate effect of the urea biosensor. A flexible arrayed RuO_2_ urea biosensor was fabricated which was measured by the voltage–time (V–T) measurement system. The experimental results showed that the RuO_2_ urea biosensor had an average sensitivity of 1.860 mV/(mg/dL) and a linearity of 0.999. By applying the voltage regulation technique, the proposed NCC reduced the drift rate of the flexible arrayed RuO_2_ urea biosensor to 0.02mV/hr. These experimental results indicate that the drift rate measured by the NCC was 98.77% lower compared to that of the conventional V–T measurement system. The drift rate measured by the proposed NCC was considerably better than the traditional V–T measurement system. Moreover, the structure of this newly proposed readout circuit is simple and is suitable to apply on different biosensors to reduce non-ideal effects.

## Figures and Tables

**Table 1:** The sensing characteristics of the flexible arrayed RuO_2_ urea biosensors over a urea concentration ranging from 10 mg/dL to 50 mg/dL.

Membrane	Urea Concentration (mg/dL)	Response Voltage (mV) (Mean ± SD)	Average Sensitivity (mV/(mg/dL))	Linearity	LOD (mg/dL)
Urease/RuO_2_	10	73.49 ± 2.06	1.860	0.999	1.345
	20	92.26 ± 3.46			
	30	111.48 ± 4.08			
	40	131.63 ± 5.96			
	50	147.14 ± 3.76			

**Table 2:** Comparisons of the sensing properties of urea biosensors with various sensing films.

Sensing membrane	Liner Range (mg/dL)	Average Sensitivity (mV/(mg/dL))	Linearity	Limit of Detection (LOD)	Reference
Urease/RuO_2_	10–50	1.860	0.999	1.345 mg/dL (0.239 mM)	This work
SnO_2_	5–80	1.980	0.976	−	[19] 2006
Magnetic microparticles-Urease/ Iridium oxide/Pt	0–7.5	1.964	0.997	78 μM	[38] 2013
Preparation, characterization, and application of urease nanoparticles for construction of an improved potentiometric urea biosensor	0.012–0.480	1.001	0.999	1 μmol/L	[39] 2018
Urease-MBs/GO/NiO	10–50	6.343	0.960	1.338 mg/dL (0.223 mM)	[24] 2019
Urease/TiO_2_	10–50	1.445	0.977	−	[27] 2019
Urease/RuO_2_	10–50	1.220	0.956	−	[34] 2019

**Table 3:** Comparison of drift rates of urea biosensor with different sensing membranes.

Sensing Membrane	Drift Rate (mV/hr)	Reference
RuO_2_ measured by NCC (the proposed new calibration circuit)	0.02	This work
RuO_2_ measured by (the V–T measurement system)	1.623	This work
Urease-MBs/GO/NiO	1.551	[24] 2019
MBs-Urease/GO/TiO_2_	4.395	[27] 2019
